# Combined Effects of Temperature and Salinity Affect the Surviv-Al of Asian Green Mussel (*Perna viridis*) through Digestive and Antioxidant Performance

**DOI:** 10.3390/antiox11102009

**Published:** 2022-10-11

**Authors:** Zhenhua Ma, Zhengyi Fu, Jingru Yang, Gang Yu

**Affiliations:** 1 Tropical Aquaculture Research and Development Center, South China Sea Fisheries Research Institute, Chinese Academy of Fishery Sciences, Sanya 572426, China; 2 Key Laboratory of Efficient Utilization and Processing of Marine Fishery Resources of Hainan Province, Sanya Tropical Fisheries Research Institute, Sanya 572018, China; 3 College of Science and Engineering, Flinders University, Adelaide 5042, Australia

**Keywords:** molluscs, tropical, water environment, water parameters, response surface, MANOVA

## Abstract

Changes in temperature and salinity of the marine environment fluctuate continuously, and the effects of these changes on shellfish survival are significant. In this study, the survival rate of adult Asian green mussels (*Perna viridis*) was measured after short-term treatments (7 days) for a range of temperature (20 °C, 25 °C, 30 °C) and salinity (23‰, 28‰, 33‰). The digestive (amylase, lipase, trypsin and pepsin activities) and antioxidant performance (SOD and GPX gene expression; SOD, GPX and CAT activities; GSH and MDA Concentration) were measured and MANOVA results were obtained using a generalized linear model between certain factors (temperature (T), salinity (S) and temperature × salinity (T × S)) and survival rate. Both T and T × S significantly affected the survival rate of Asian green mussels. The overall relationship between the survival rate of *P. viridis* and T and S within the experimental range can be summarised by the equation: ln(Survival rate) = 54.9282 − 2.5627 × T − 3.6180 × S + 0.1857 × T × S + 0.0156 × T2 + 0.0520 × S2 − 0.0012 × T2 × S − 0.0023 × T × S2. The optimal temperature–salinity combination was 23.698 °C/30.760‰. T, S and T × S all had a significant impact on amylase and lipase activities of Asian green mussels. In the hepatopancreas, gill, mantle, adductor muscle, gonad and foot tissues of Asian green mussel, the SOD and GPX gene expression were responsive to temperature–salinity changes. The antioxidant performance of the combinations far from the optimal temperature–salinity combination were significantly improved. Our results suggest that combined temperature and salinity effects have a regular impact on the survival of Asian green mussels and that there is a link between survival and digestive and antioxidant performance.

## 1. Introduction

The Asian green mussel (*Perna viridis*) is a large, fast-growing marine bivalve mollusc widely distributed in coastal and estuarine areas of the Asian-Pacific regions [1]. Due to its fast growth rate and high fecundity, it has excellent cultural potential in tropical coastal areas of Asia [2,3]. The Asian green mussel is also a popular delicacy around the world, as it is commonly found in seafood risotto and French cuisine and is also processed into dried products for storage in the southern coast of China. The Asian green mussel is not only tasty but also highly nutritious, and it is harvested as a food source due to its high levels of vitamins and minerals [4]. They are considered a cheap source of protein for people living in coastal regions as they contain 36.15% protein [5,6]. In addition, the Asian green mussel is a widely used as an ecological marker because of its wide distribution and filter feeding characteristics, making it useful for monitoring environmentally harmful substances such as microplastics, heavy metals and pesticides [7,8,9]. On the other hand, because of its rapid growth and environmental tolerance, Asian green mussel has become an invasive species in some areas [1]. At the same time, its massive expansion may damage submerged structures such as drainage pipes and navigational buoys.

Based on the bifacial nature of the Asian green mussel, it is essential to study its ability to survive under environmental changes. It can better serve to increase aquaculture production but also help to manage its biological invasion. In the marine environment, temperature and salinity fluctuate from time to time due to weather, tides, mixing, river inflow, etc., which is why they are the primary factors considered in most studies on the effects of environmental changes on the survival of marine organisms. Especially in Bivalvia, the filter-feeding nature is so entirely in contact with the water environment that its survival is very much influenced by the environment [10,11]. In Asian green mussels, there are some studies on the effects of single factors of temperature and salinity on survival [12,13,14,15]. There are also some studies on the combined effect of temperature and salinity. For example, Yuan’s study investigated the temperature and salinity tolerance range of juvenile and adult Asian green mussels and the combined effect of temperature and salinity on survival [1]. Currently, no studies have been conducted on adult Asian green mussels to address changes in the tropical water environment that try to explain the causes of survival changes. 

The physiological functions of aquatic animals are often influenced by the water environment, and altered physiological states in an unsuitable environment can often affect health or even lead to death [16,17,18,19]. Changes in the environment can affect the feeding behaviour of shellfish, and studies have shown that high water temperatures can negatively affect the feeding and digestive processes of *Mytilus galloprovincialis* [20]. In the long term, this can affect the mussels’ nutritional intake, impacting their health. In addition, environmental changes are prone to induce changes in the antioxidant capacity of aquatic animals. In the bivalve shellfish *Chlamys farreri*, *Corbicula japonica*, *Perna canaliculus*, *Pinctada fucata martensii*, *Ruditapes decussatus*, *R. philippinarum* and *Solenaia oleivora*, there are corresponding response mechanisms in the face of oxidative stress states caused by drastic changes in temperature or salinity [21,22,23,24,25,26,27]. Gene expression regulation is probably one of the most rapid and sensitive responses to environmental stress, and there have been many examples of gene expression changes in response to environmental stress, as well as many studies that have used key genes such as SOD as important indicators for evaluating the oxidative stress response [28,29]. In this study, the combined effects of temperature and salinity in the tropics on the survival rate of adult Asian green mussel were investigated with reference to the range of water temperature and salinity in the South China Sea, and an attempt was made to explain the changes in survival rate in terms of changes in digestive and antioxidant performances. This study is intended to provide a reference for the culture of Asian green mussel and their flooding management.

## 2. Materials and Methods

### 2.1. Animals

The *P. viridis* were collected from Xincun Bay, Lingshui Town, Hainan, China (18°24′14.7″ N 110°00′24.1″ E) and transported to the Tropical Aquaculture Research and Development Center, South China Sea Fisheries Research Institute, Chinese Academy of Fishery Sciences (Lingshui Town, Hainan, China). After removing the attached organisms on the shell surface, healthy individuals of a similar size (body mass: 37.44 ± 3.85 g, shell length: 7.91 ± 0.46 mm) were randomly assigned to tanks (300 L) and acclimated for a week. During the acclimation, the environmental parameters were maintained at salinity 33 ± 1‰, temperature 25 ± 1 °C, ammonia nitrogen < 0.1 mg/L, nitrite nitrogen <0.02 mg/L, pH 8.0, dissolved oxygen > 7.0 mg/L and light intensity < 500 Lx with a natural photoperiod. *Platymonas subcordiformis* (2 × 10^5^ cells/mL) was fed daily (9:00–9:30) in the morning, and 50% of the seawater was replaced daily. Half of the seawater was replaced, feces and residues were siphoned off daily, and dead shellfish were removed from the tank immediately during acclimation and experiment.

### 2.2. Experiment Design

A complete 3 × 3 factorial design was employed, and variables included temperature and salinity. Preliminary trials, based upon the seasonal changes in salinity and temperature of the seawater in the South China Sea, were conducted to define the settings of salinity and temperature chosen for the combination experiment. Values for temperature ranged from 20 °C to 30 °C, and values for salinity ranged from 23‰ to 33‰ (Table 1). There were nine salinity–temperature combined treatments in the experiment; each treatment had three replicates and each replicate contained 15 *P. viridis*. The experiment lasted 5 days. The temperature of the experimental tanks was gradually adjusted from 28 °C to the temperature corresponding to each treatment at 1 °C per 1 h (Hostins et al., 2015). The salinities were adjusted 4–6‰ daily until the salinity in each tank reached the desired level (Esparza-Leal et al., 2016). The water temperature of experiments was manipulated by heating rods or ice bottles. All salinities were prepared by adding sea salts or tap water with 24 h aeration to the natural double-filtered seawater. A salinometer (ATAGO S-10E) was used to gauge the salinity. Except for temperature and salinity, the other environments and culture procedures in the treatments were kept the same during acclimation.

### 2.3. Sampling

Three *P. viridis* were randomly collected from each tank, and the hepatopancreas, gill, mantle, adductor muscle, gonad and foot tissues were cut off on an ice tray with scissors. Rinsed with pre-cooling 0.9% normal saline, blotted with clean filter paper, the tissue samples were quickly placed in 2 mL centrifuge tubes and stored at −80 °C. The biochemical parameters of hepatopancreas were determined according to the manufacturer’s instructions using commercial kits (Nanjing Jiancheng Bioengineering Institute, Nanjing, China), i.e., amylase: iodine-starch assay method; lipase: 6′ Methylresorufin method; trypsin: N-α-Benzoyl-L-arginine ethyl ester method; pepsin: albumin-bromphenol blue method; Superoxide Dismutase (SOD): hydroxylamine method; Glutathione Peroxidase (GPX): enzymatic method; Catalase (CAT): ammonium molybdate method; Reduced glutathione (GSH): 2-nitrobenzoic acid (DTNB) method; Malondialdehyde (MDA): thiobarbituric acid (TBA) method. The protein concentration of the enzyme extracts was measured using the Bradford method (Bradford, 1976). All biochemical parameter analyses were performed in triplicate.

### 2.4. Gene Expression

The harvested frozen tissues were homogenized in 1 mL Trizol (Invitrogen, Waltham, MA, USA) on ice using a hand-holding homogenizer (Greenprima Instrumenta Co., Ltd., London, UK). Total RNA was extracted according to the manufacturer’s instructions. The concentration of the RNA was quantified by spectrophotometry (Bioteke Corporation Co., Ltd., Beijing, China). Finally, the integrity of RNA was assessed using agarose gel electrophoresis. The RNA was immediately used for cDNA synthesis. Subsequently, reverse transcription was performed on 1 μg of total RNA using TransScript-Uni One-Step gDNA Removal and cDNA Synthesis SuperMix (Transgen Biotech Co., Ltd., Beijing, China). The synthesized cDNA samples were stored at −20 °C until further use.

The qPCR was performed with the Real-time qPCR analysis (Analytik Jena GmbH, Jena, Germany) using SYBR Green (Tiangen Biotech Co., Ltd., Beijing, China). The primers of SOD, GPX and β-actin were previously designed and validated by Ref (Table 2). The 20 μL of reaction, including 10 μL 2 × RealUniversal PreMix, 0.6 μL of each primer (10 μM) and 2 μL of diluted cDNA was initially denatured at 95 °C for 15 min and then amplified for 40 cycles (95 °C, 10 s, 58 °C, 20 s and 72 °C, 30 s). At the end of each qRT- PCR cycle, the melting curve analysis of the primers was performed to ensure only specific products were obtained with no formation of primer dimers. No template control was included with each assay to verify that PCR master mixes were contamination-free. The relative mRNA expression levels of the target genes were determined by the 2^−ΔΔCt^ method and were normalized based on the level of the housekeeping gene (β-actin). The reaction efficiency was 90–110%, and Pearson’s coefficients of determination (R^2^) > 0.97.

### 2.5. Response Surface Analysis of Temperature-Salinity Interaction

The respiratory survival rate was assessed utilizing the Central Composite Design (CCD) with either 2-factors or explanatory variables, temperature (T, °C) and salinity (S, ‰) [32]. The mathematical response model developed was in the following form:

$$y=\beta_{0}+\overset{k}{\underset{i=1}{\sum }}\beta_{i}x_{i}+\overset{k}{\underset{i=1}{\sum }}\beta_{ii}x_{i}^{2}+\overset{k-1}{\underset{i-1}{\sum }}\overset{k}{\underset{j=2}{\sum }}\beta_{ij}x_{i}x_{j}+\varepsilon$$

where *y* is the predicted response, *β_o_* is a constant term coefficient, *β_i_* is a linear term, *β_ii_* is a quadratic term, *β_ij_* is an interaction term, ε is an error term and *x_i_x_j_* is the coded values of the independent variables. After the model equation was established, the optimization program of Design Expert 12 software was used to optimize and analyze several responses. By maximizing the conversion of the expected function, the optimal condition set was finally obtained.

### 2.6. Statistical Analysis

The data were expressed as the mean ± standard deviation (SD). Statistical analyses were carried out by PASW Statistics (version 18). Spearman correlation tests were used to assess the relationships between the single factors (temperature and salinity) and the survival rates of *Perna viridis*. Multiway within-subjects MANOVA (repeated measure) was used to analyze the data from bioassays that examined the combined effects of temperature and salinity on *P. viridis*. Again due to a strong interaction among the factors, the effect of each factor was tested at a fixed level of the other factor(s) using one-way ANOVA and Tukey’s multiple comparison test. Differences were analyzed by repeated measure ANOVA followed by Tukey’s multiple comparison test.

## 3. Results

### 3.1. Survival Rate

Under the experimental range conditions, the temperature had a significant effect on the survival of *Perna viridis* (*p* < 0.01), while salinity had no significant impact (*p* > 0.05). However, the interaction of temperature × salinity on survival was significant (*p* < 0.01, Table 3).

**Figure 1:**
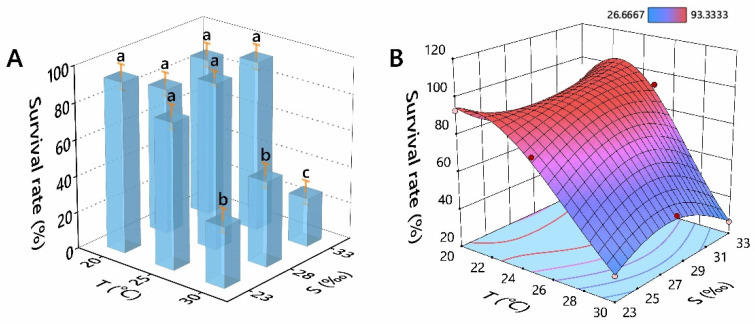
Effects of temperature and salinity on the survival rates of *Perna viridis.* ((**A**) histogram; (**B**) response surface plot). Different letters indicate significant differences (*p* < 0.05).

Survival rates showed a significant difference between treatments, especially in temperature (*p* < 0.05, Figure 1A). Survival rates were significantly lower in the different salinity treatments at 30 °C than at 20 °C and 25 °C. At 30 °C, survival rates were significantly higher in the lower salinities (23‰ and 28‰) than in the control salinity (33‰).

The linear correlation between single factor and survival rate was tested. The Spearman correlation coefficient between temperature and survival rate was a significant negative correlation (*p* < 0.01), and the Spearman correlation coefficient between survival rate and salinity showed no significant correlation (*p* > 0.05, Table S1). To investigate the combined effect of temperature and salinity on survival rate, the construction of the corresponding surfaces was carried out (Figure 1B). The overall relationship between the survival rate of *P. viridis* and temperature(T) and salinity(S) within the experimental range can be summarised by the equation:

ln(Survival rate) = 54.9282 − 2.5627 × T − 3.6180 × S + 0.1857 × T × S + 0.0156 × T^2^ + 0.0520 × S^2^ − 0.0012 × T^2^ × S − 0.0023 × T × S^2^. The ANOVA for reduced cubic model is shown in Table S1. Within the experimental range, the optimal temperature/salinity combination is 23.698 °C/30.760‰, at which survival rate (99.999%) peaks, with the desirability being 100% (Table S2).

### 3.2. Digestive Enzymes

**Figure 2:**
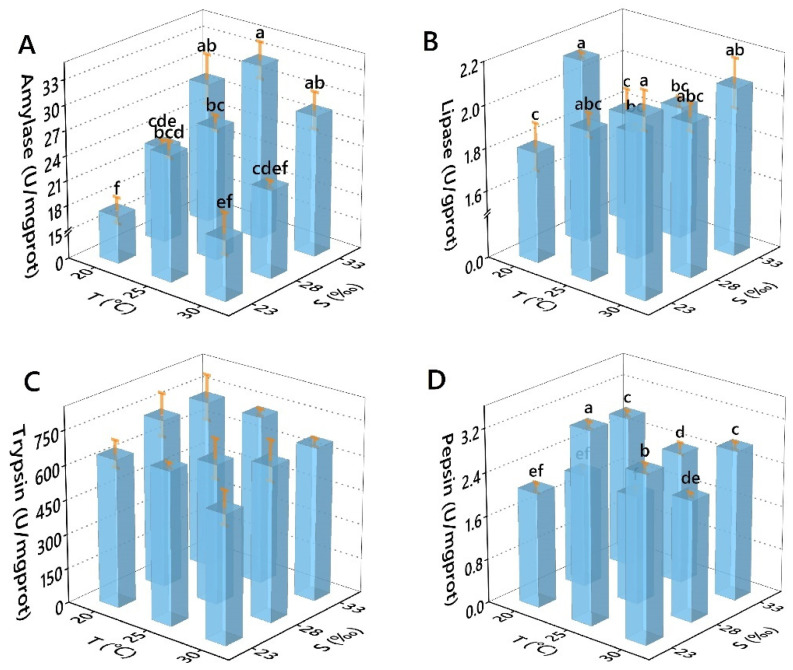
Effects of temperature and salinity on the digestive enzyme activities. ((**A**) Amylase, (**B**) Lipase; (**C**) Trypsin; (**D**): Pepsin) of *Perna viridis.* Different letters indicate significant differences (*p* < 0.05).

Both single factor and combined effects of salinity and temperature significantly impacted digestive enzyme activities, except S-lipase and all (S, T, T × S)-trypsin (Table 4). In the experimental range, the activity of amylase increased with the increase of salinity, increased first and then decreased with the rise in temperature and reached the peak at 25 °C/33‰ (Figure 2A). However, lipase activity in the experimental range showed a trend of increasing at the edge temperature/salinity and decreasing in the middle temperature/salinity. The highest and lowest values of activity appeared at 20 °C, and the highest values were 20 °C/28‰ (Figure 2B). Trypsin activity showed no significant differences between the groups (Figure 2C). Pepsin activity showed irregular fluctuation in the experimental range, and the highest value was 25 °C/23‰ in all salinity levels (Figure 2D).

### 3.3. Anti-Oxidation Performance

**Figure 3:**
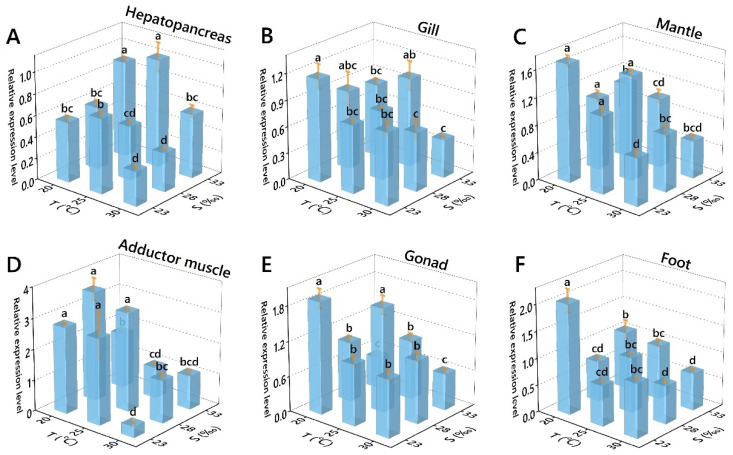
Effects of temperature and salinity on the SOD relative expression level of *Perna viridis* in different tissues. ((**A**) Hepatopancrear; (**B**) Gill; (**C**) Mantle; (**D**) Adductor muscle; (**E**) Gonad; (**F**) Foot). Different letters indicate significant differences (*p* < 0.05).

Both single factors and combined effects of salinity and temperature significantly impacted the relative expression level of SOD (Table 5). The relative expression levels of SOD in hepatopancreas increased and then decreased with increasing temperature and increased with increasing salinity, with the maximum value occurring at 25 °C/33‰ and the minimum value at 30 °C/23‰ (Figure 3A). The relative expression levels of SOD in gills decreased with increasing temperature and salinity, except at 25 °C/33‰ where there was an outward trend of increase, with the maximum value occurring at 30 °C/23‰ and the minimum value at 30 °C/33‰ (Figure 3B). The relative expression levels of SOD did not show a clear trend in the mantle, with the maximum value at 20 °C/23‰ and the minimum value at 30 °C/23‰ (Figure 3C). The relative expression levels of SOD in the adductor muscle decreased with increasing temperature, with no clear trend in the different salinities, with the maximum value occurring at 20 °C/28‰and the minimum value at 30 °C/23‰ (Figure 3D). There was no significant trend in the relative expression levels of SOD in the gonad and foot; the maximum value occurred at 20 °C/23‰, the minimum value occurred at 20 °C/33‰ in the gonad and occurred at 25 °C/23‰ in foot (Figure 3E,F).

**Figure 4:**
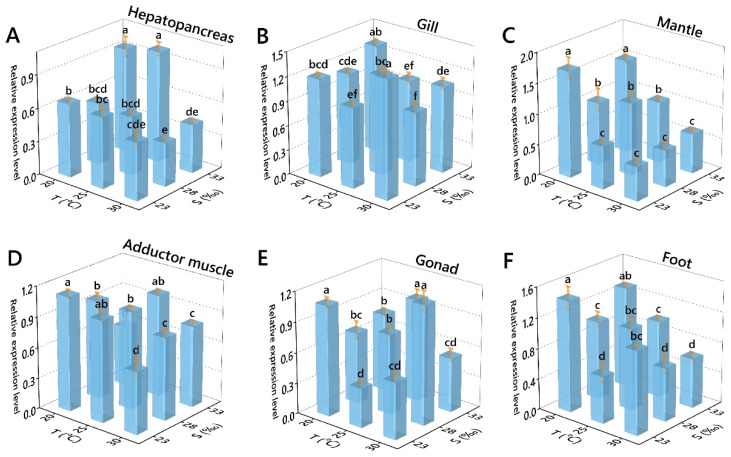
Effects of temperature and salinity on the GPX relative expression level of *Perna viridis* in different tissues. ((**A**) Hepatopancrear; (**B**) Gill; (**C**) Mantle; (**D**) Adductor muscle; (**E**) Gonad; (**F**) Foot). Different letters indicate significant differences (*p* < 0.05).

Both single factor and combined effects of salinity and temperature significantly impacted the relative expression level of GPX, except for salinity-mantle (Table 6). The relative expression levels of GPX in hepatopancreas decreased with increasing temperature. They decreased then increased with increasing salinity, with the maximum value occurring at 25 °C/33‰ and the minimum value at 30 °C/28‰ (Figure 4A). The relative expression levels of GPX in gills increased then decreased with increasing temperature and salinity, except for 25 °C/28‰, where there was an outward trend of increase, with the maximum value occurring at 30 °C/23‰ and the minimum value at 30 °C/28‰ (Figure 4B). The relative expression levels of GPX in the mantle decreased with increasing temperature. At the same time, there was no significant trend in different salinities, with the maximum value at 23 °C/23‰ and the minimum value at 30 °C/23‰ (Figure 4C). There was no significant trend in the relative expression levels of GPX in the adductor muscle, with the maximum value occurring at 20 °C/23‰ and the minimum value at 30 °C/23‰ (Figure 4D). The relative expression levels of GPX in gonad and foot showed no significant trend, with the maximum value occurring at 20 °C/23‰ and the minimum value at 20 °C/33‰ (Figure 4E,F).

**Figure 5:**
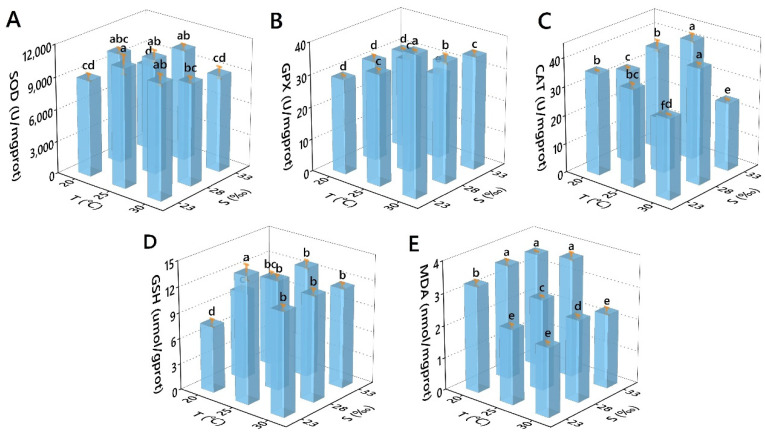
Effects of temperature and salinity on the antioxidant-related indicators. ((**A**) SOD; (**B**) GPX; (**C**) CAT; (**D**) GSH; (**E**) MDA) of *Perna viridis.* Different letters indicate significant differences (*p* < 0.05).

In the hepatopancreas of *Perna viridis*, both single factor and combined effects of salinity and temperature significantly impacted antioxidant-related biochemical indicators (Table 7). SOD activity showed a trend of rising and then falling with the increase in temperature. There was no obvious pattern in different salinities, with the maximum value appearing at 25 °C/23‰ and the minimum value appearing at 20 °C/33‰ (Figure 5A). GPX activity increased with the increase of temperature and decreased with the increase of salinity, with the maximum value emerging at 30 °C/23‰ and the minimum value appearing at 25 °C/33‰ (Figure 5B). There was no obvious pattern in CAT activity with temperature and salinity, with the maximum value occurring at 30 °C/28‰ and the minimum value at 25 °C/28‰ (Figure 5C). GSH concentration increased and then decreased with temperature, with no apparent pattern in different salinities, with the maximum value at 25 °C/23‰ and the minimum value at 20 °C/23‰ (Figure 5D). MDA concentration decreased with temperature, with no apparent pattern in different salinities, with the maximum value occurring at 25 °C/33‰ and the minimum value occurring at 30 °C/23‰ (Figure 5E).

## 4. Discussion

Although the Asian green mussel is a shellfish that can survive in the tropics, it seems to prefer colder water. In this study, elevated temperatures seriously negatively impacted Asian green mussel survival. The optimum temperature for Asian green mussels from Guayacan Venezuela in the open-flow system is 20.1–29.1 °C, with an optimum temperature of 26 °C [33]. In Marudu Bay, Malaysia, the optimum temperature for Asian green mussels is 26–32 °C [34]. In this study, the survival rate of 20 °C and 25 °C remained significantly higher, and the optimum temperature of Asian green mussels seems to be lower than in the areas mentioned above. The results of these studies also suggest that the Asian green mussel appears to have the potential to adapt to the climate of different regions. The Asian green mussels from the southeastern United States can survive in a wide range of temperatures (9–35 °C); however, as salinity decreases, the thermal survival range becomes narrower [1]. In the present study, a slight decline in salinity did not seem to have a negative effect on the thermal survival of Asian green mussels. The reason for the disagreement is that Yuan’s conclusions were obtained over a range of experimental conditions from 0 ppt–40 ppt. In the present study, due to the small range of experimental conditions for salinity, the distribution of salinity in the set of optimal solutions obtained was spread over the entire range of experimental conditions, while the effect of single-factor salinity on survival was not significant. The salinity range of this experiment was determined by the fluctuation of salinity in the South China Sea due to rainfall and other weather conditions, which shows that the salinity fluctuation in this natural condition has little effect on the survival of Asian green mussels. Differences in temperature and salinity for optimal survival rate of Asian green mussel in different studies can also be explained due to differences in body size. Larger mussels were more tolerant to environmental changes than smaller mussels [1,35]. The Asian green mussels of this study were adults around 70 mm, and information about comparing the environmental tolerance of larvae, juveniles and adults is to be added in future studies.

The digestive performance of aquatic organisms is easily influenced by their environment, especially molluscs [36,37]. In addition to influencing the feeding behavior of filter-feeding molluscs, the environment also affects their digestive enzyme activity [38,39]. In the present study, the amylase showed a significant pattern of change. The results of this study had a similar trend to the results of *P. viridis* in estuarine Ma Wan, Hong Kong [40]. The reasons for this trend are likely different, in that Asian green mussel in Ma Wan survive in a natural environment where their digestive enzymes are influenced by changes in food conditions and not by temperature, but in this study was conducted under laboratory conditions with strict control of other variables, so we assume that changes in Asian green mussel amylase are temperature-dependent when other disturbances are excluded. On the one hand, in in vitro assays, shellfish amylase activity changes with temperature and has an optimum temperature [41]; on the other hand, water temperature changes its feeding behavior, which leads to changes in algal intake [42]. At present, there is no direct reason to correlate changes in amylase activity with salinity, but it has been shown that *Corbicula japonica* at unsuitable salinities will consume large amounts of food to supplement energy to reduce oxidative stress when there is sufficient food in the water [24]. However, in this study, because the intolerable salinity of Asian green mussel was not reached, the increase in amylase activity due to high food intake was not reflected in the relatively unsuitable salinity. In the current research, lipase showed some regularity of change with temperature variation. Evidence from in vitro experiments that the lipase activity of invertebrates varies with temperature [43,44].

In aquatic animals, SOD and GPX are the first to respond to the antioxidant system [45,46]. In this study, there was no uniform pattern of SOD across tissue salinity, but basically, low temperature promoted transcription. The pattern was not as clear as that of SOD in the relative expression levels of GPX across tissues. Still, there was a trend toward low temperature-promoted transcription as well. From this, it can be inferred that the antioxidant gene expression in all tissues responds to environmental changes. The hepatopancreas is an essential organ for digestion and immunity in molluscs and is very active in carrying out physiological reactions [47]. Among these indicators characterizing antioxidant performance, GPX and GSH are mainly promoted by high temperatures. GPX uses GSH as a cofactor to catalyze the conversion of H_2_O_2_ and organic hydrogen peroxide into water and alcohol. Thus, the increased GPX activity observed in mussels exposed to elevated temperatures may be a protective cellular mechanism [48]. These changes in antioxidant enzymes and antioxidants lead to efficient clearance of MDA. MDA is the most abundant aldehyde produced and indicates lipid peroxidation in the biological system [49].

Based on our experience with the non-fluvial culture of bivalve shellfish under laboratory conditions, survival is not guaranteed in the long term, even under optimal conditions, a situation in which changes in environmental factors magnify the effect on survival even more. In natural environments, such low survival rates do not occur even when the less suitable temperature–salinity combinations of the present experimental conditions are reached [6]. From an antioxidant perspective, the unsuitable temperature-salinity combinations improved antioxidant performance and effectively scavenged malondialdehyde, indicating that the antioxidant system of Asian green mussel responded effectively to combined temperature-salinity changes. However, inappropriate temperature-salinity combinations weakened some digestive performance from the perspective of digestive enzymes. This could potentially lead to an inadequate energy supply to its organism, which, combined with an active antioxidant system [50], could weaken other physiological functions and therefore threaten the health of Asian green mussels. There is also a hypothesis that since our samples for the determination of physiological and biochemical indicators came from surviving Asian green mussels, in some unsuitable temperature-salinity combinations, these surviving individuals were in the overall minority and were screened out by the environment to eliminate individuals with a weak antioxidant system response, resulting in a more robust antioxidant performance response in biochemical indicators for the unsuitable temperature-salinity combinations. Such differences are common even in the same batch of aquatic animals [51]. In this study, changes in survival can be explained in terms of digestive and antioxidant performance in the combined temperature–salinity effect. However, it is still expected that changes in the survival of Asian green mussels can be explained in terms of other physiological functions in future studies.

## 5. Conclusions

In conclusion, the overall relationship between the survival rate of *P. viridis* and T and S within the experimental range (temperature: 20–30 °C; salinity: 23–33‰) can be summarised by the equation: ln(Survival rate) = 54.9282 − 2.5627 × T − 3.6180 × S + 0.1857 × T × S + 0.0156 × T2 + 0.0520 × S2 − 0.0012 × T2 × S − 0.0023 × T × S2. The optimal temperature–salinity combination was 23.698 °C/30.760‰. Both single factor and combined effects of salinity and temperature significantly impacted digestive enzyme activities, the relative expression level of SOD and GPX, antioxidant-related biochemical indicators, except S-lipase, all (S, T, T × S)-trypsin and S-mantle of the relative expression level of GPX.

## Figures and Tables

**Table 1:** Setup of experimental treatments for temperature and salinity.

Treatment	Temperature (°C)	Salinity (‰)
1	20	23
2	20	28
3	20	33
4	25	23
5	25	28
6	25	33
7	30	23
8	30	28
9	30	33

**Table 2:** The primer pairs used in this study.

Gene Name	Primer Sequence 5′-3′	Accession Number	References
SOD	F: GCAACATTCCTTCAGCACCT	AJ496219.1	He et al. (2019) [30]
	R: CCTTGTTCCAAAAGCCTAATTG		
GPX	F: CAACGACCCCCAGATTCAGA	HQ891311.1	He et al. (2019) [30]
	R: TCTAGAGTCGGTAGGAGCCAT		
β-actin	F: ACTCCGTCTGGATTGGTG	AGZ84261	Wang et al. (2017) [31]
	R: CTCGTCGTATTCTTGTTTGC		

**Table 3:** MANOVA results using a generalized linear model for the main and interaction effects of temperature and salinity on *Perna viridis* survival rates.

Source	*df*	MS	F	*p*
T	2	8047.74	193.54	0.001
S	2	40.35	0.97	0.398
T × S	4	277.25	6.67	0.002
Error	18	41.58		

**Table 4:** MANOVA results using a generalized linear model for the main and interaction effects of temperature and salinity on *Perna viridis* digestive enzymes.

Source	*df*	Amylase		Lipase		Trypsin		Pepsin	
		MS	F	MS	F	MS	F	MS	F
T	2	89.26	38.71 **	0.06	11.36 **	10,285.39	2.13	0.36	63.91 **
S	2	196.07	85.03 **	0.02	2.69	16,421.87	3.40	1.53	273.49 **
T × S	4	6.93	3.01 *	0.06	10.47 **	6890.96	1.43	0.69	124.06 **
Error	18	2.31		0.01		4827.08		0.01	

* *p* < 0.05, ** *p* < 0.01.

**Table 5:** MANOVA results using a generalized linear model for the main and interaction effects of temperature and salinity on the SOD relative expression level in *Perna viridis*.

Source	*df*	Hepatopancreas		Gill		Mantle		Adductor Muscle		Gonad		Foot	
		MS	F	MS	F	MS	F	MS	F	MS	F	MS	F
T	2	0.25	89.45 **	0.24	21.67 **	0.94	216.89 **	7.77	104.50 **	0.34	37.10 **	0.57	59.17 **
S	2	0.33	117.33 **	0.07	6.21 **	0.24	54.71 **	4.46	59.94 **	1.01	109.39 **	0.50	52.01 **
T × S	4	0.02	6.64 **	0.10	8.68 **	0.24	54.93 **	1.38	18.55 **	0.59	63.63 **	0.52	54.50 **
Error	18	0.00		0.01		0.00		0.07		0.01		0.01	

** *p* < 0.01.

**Table 6:** MANOVA results using a generalized linear model for the main and interaction effects of temperature and salinity on the GPX relative expression level of *Perna viridis*.

Source	*df*	Hepatopancreas		Gill		Mantle		Adductor Muscle		Gonad		Foot	
		MS	F	MS	F	MS	F	MS	F	MS	F	MS	F
T	2	0.22	100.58 **	0.05	16.44 **	1.55	145.15 **	0.14	52.49 **	0.04	8.53 **	0.52	62.19 **
S	2	0.21	93.48 **	0.04	14.01 **	0.03	3.22	0.04	14.21 **	0.10	22.23 **	0.04	4.23 *
T × S	4	0.05	23.92 **	0.14	47.81 **	0.28	26.17 **	0.11	42.11 **	0.36	79.85 **	0.23	27.71 **
Error	18	0.00		0.00		0.01		0.00		0.00		0.01	

* *p* < 0.05, ** *p* < 0.01.

**Table 7:** MANOVA results using a generalized linear model for the main and interaction effects of temperature and salinity on the antioxidant-related indicators of *Perna viridis*.

Source	*df*	SOD		GPX		CAT		GSH		MDA	
		MS	F	MS	F	MS	F	MS	F	MS	F
T	2	6,161,165.16	27.44 **	179.02	350.44 **	2,907,263.01	14.90 **	38.06	165.02 **	2.89	501.54 **
S	2	3,379,184.90	15.05 **	64.22	125.71 **	3.97	3.94 *	0.15	0.65 *	0.81	141.17 **
T × S	4	1,517,985.18	6.76 *	30.08	58.89 **	14.07	62.26 **	5.17	22.4 **	0.40	69.68 **
Error	18	224,555.28		0.51		1.01		0.23		0.01	

* *p* < 0.05, ** *p* < 0.01.

## Data Availability

The data presented in this study are available on request from the corresponding author. The data are not publicly available due to they relate to data that has not yet been published.

## Supplementary Materials

The following supporting information can be downloaded at: https://www.mdpi.com/article/10.3390/antiox11102009/s1. Table S1: ANOVA for Reduced Cubic model; Table S2: Optimal set of solutions for the survival rate of Asian green mussel.
